# Hysteroscopic resection as a safe minimally invasive technique for the management of cornual pregnancy: A case report and literature review

**DOI:** 10.1002/ccr3.8137

**Published:** 2023-11-08

**Authors:** Nikolaos Tsagias, Emmanouil M. Xydias, Apostolos C. Ziogas, Panagiotis Tsikouras, Nikolaos Patsinakidis, Angelos Daniilidis, Elias Tsakos

**Affiliations:** ^1^ EmbryoClinic IVF Thessaloniki Greece; ^2^ Faculty of Medicine, School of Health Sciences University of Thessaly Larissa Greece; ^3^ Department of Medicine Democritus University of Thrace Alexandroupoli Greece; ^4^ Department of Obstetrics and Gynecology General University Hospital of Alexandroupoli Alexandroupoli Greece; ^5^ Gynecological Imaging YGEIA Radiology Center Ptolemaida Greece; ^6^ First University Department of Obstetrics and Gynecology, School of Medicine Papageorgiou General Hospital, Aristotle University of Thessaloniki Thessaloniki Greece

**Keywords:** cornual ectopic pregnancy, fertility sparing treatment, MRI, operative hysteroscopy, uterine evacuation

## Abstract

Hysteroscopic resection of ectopic cornual pregnancy following MRI imaging is a safe and effective treatment option without significantly impacting fertility potential or increasing the risk of future obstetrical complications.

## Graphical abstract

Hysteroscopic identification of gestational sac implanted at the uterine cornu (A) and subsequent, successful removal (B) in a minimally invasive, fertility sparing manner; based on prior MRI findings.

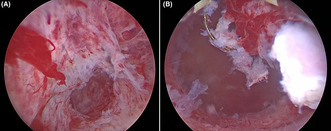

## 1 INTRODUCTION

Cornual pregnancy is rare, accounting for approximately 2–4% of all ectopic pregnancies.^1^ By definition, it refers to the implantation and development of a gestational sac at the proximal and lateral regions of the uterus (aka uterine horns or cornua). As with the majority of other types of ectopic pregnancy, cornual pregnancy diagnosis is based on clinical suspicion, β‐hCG measurements and transvaginal ultrasound findings.^2^ As with other types of ectopic pregnancy, cornual pregnancy is associated with severe patient morbidity and mortality.^3^ Therefore, timely and accurate diagnosis is key as it directly affects treatment type and urgency. Several management options are available from the conservative (methotrexate administration and expectant management) up to the more radical side of the spectrum (cornuotomy, cornual resection, hysterectomy).^1^ Hysteroscopy is an additional option that combines effective and complete removal of the gestational sac, without severely affecting uterine anatomy, however its application usually requires imaging guidance.^4^

In this report, we present the case of a cornual ectopic pregnancy, which could not be diagnosed via traditional ultrasonographic imaging and instead was verified by MRI imaging. Subsequently, based on the imaging data obtained via MRI, hysteroscopic resection and removal of all products of gestation was safely and successfully performed without any complications and with the patient making a swift recovery.

## 2 CASE PRESENTATION

**FIGURE 1:**
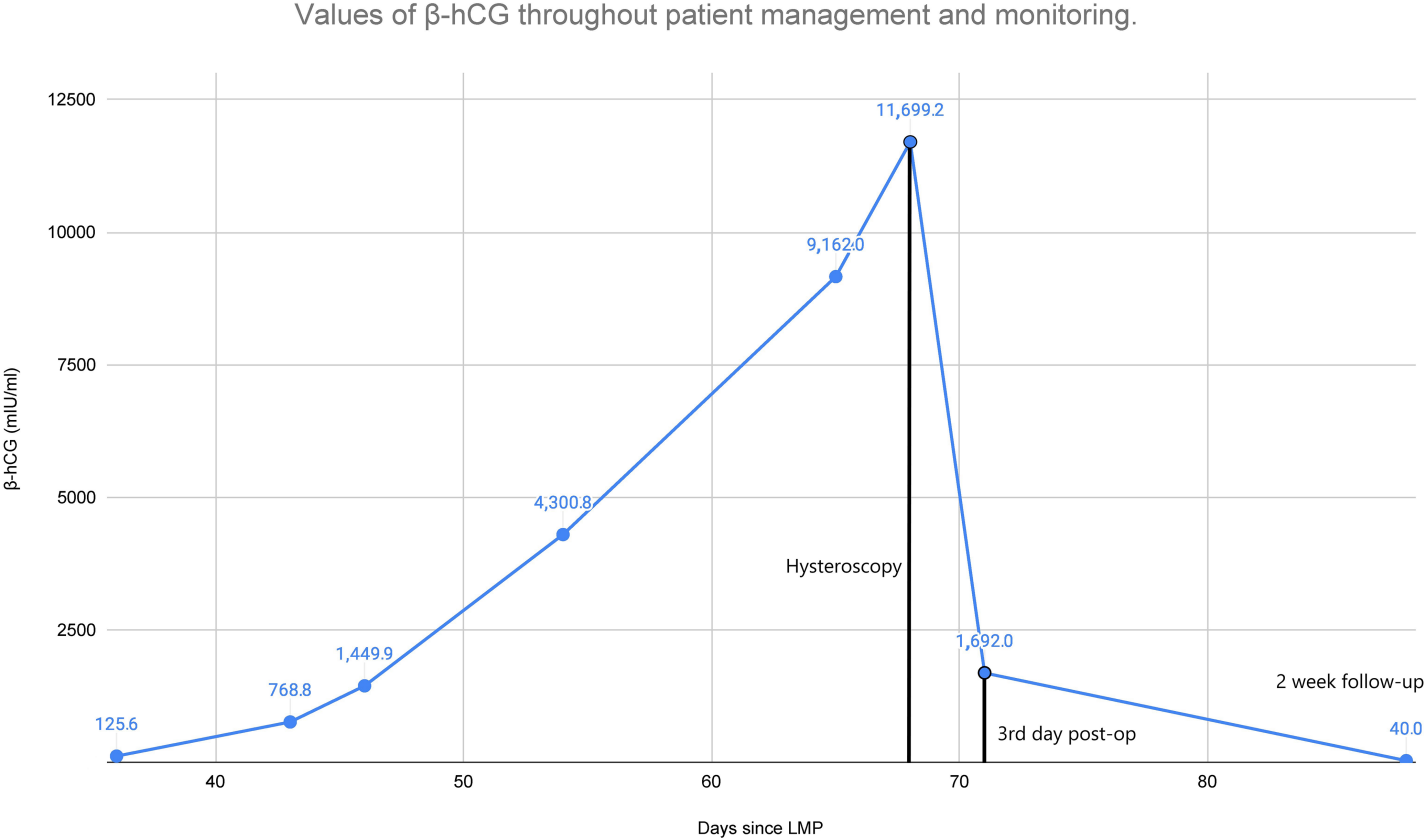
Line chart depicting the increasing levels of β‐hCG, based on repeated measurements conducted on certain intervals since the last menstrual period (LMP). There was a rapid increase of the levels pre‐operatively to the highest recorded value and subsequently a significant drop post‐operatively, denoting the successful removal of all embryonic and placental tissue.

Α 42 year old woman presented to our clinic with positive pregnancy tests seeking to initiate a routine pregnancy monitoring schedule. The levels of her serum β‐hCG were sequentially measured at regular intervals, however, they demonstrated an abnormally slow elevation pattern (Figure 1). The patient additionally mentioned vaginal bleeding and abdominal cramping during that time, thus raising clinical suspicion for further investigation. Her medical history included four instances of missed abortion, which were successfully resolved via dilation and curettage and a history of caesarean delivery of a healthy baby, complicated by massive obstetric hemorrhage, which was ultimately successfully managed. She had undergone a hysteroscopic procedure in the past, which included polypectomy and adhesiolysis in the context of fertility enhancement surgery.

**FIGURE 2:**
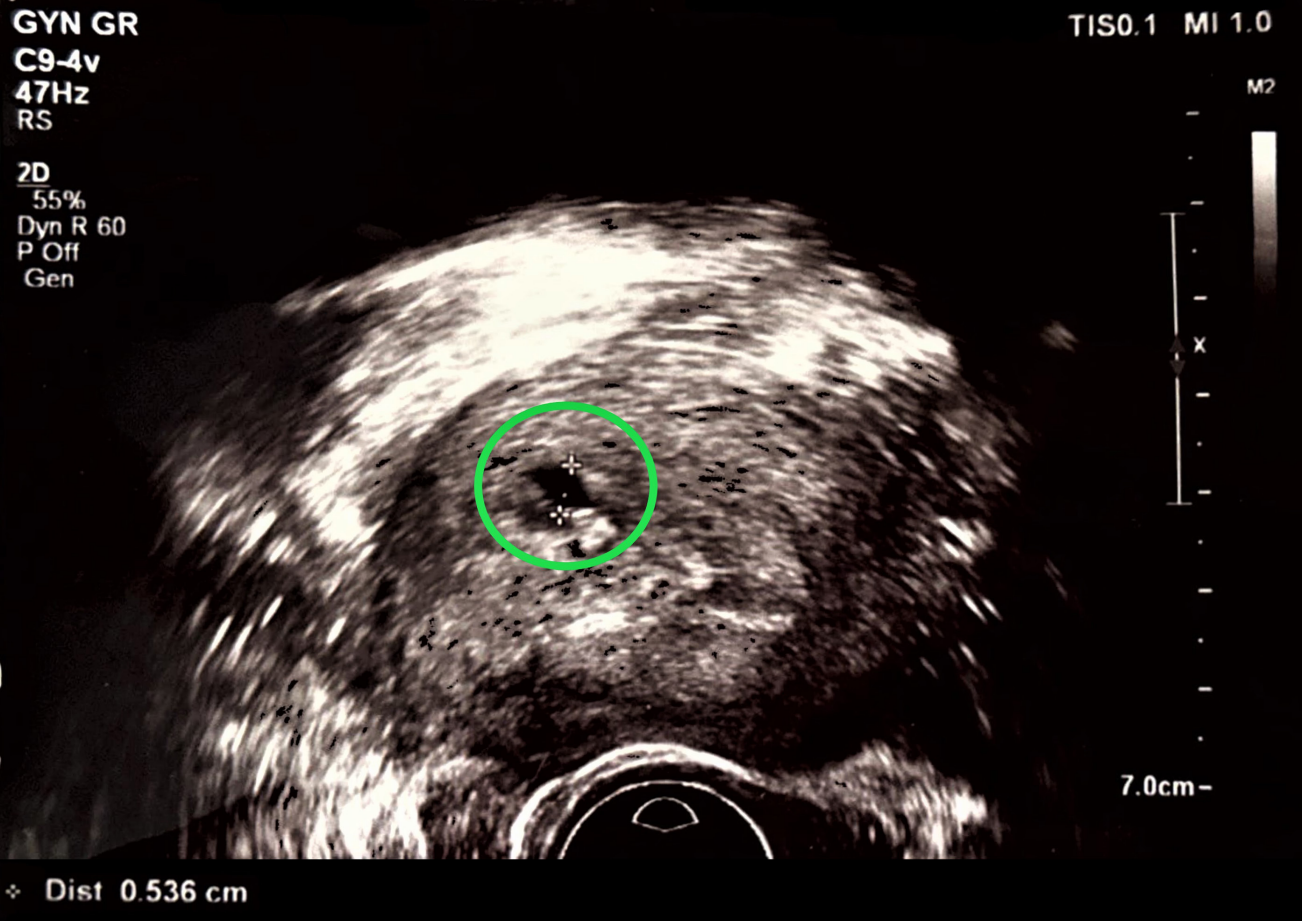
Transvaginal ultrasound findings from the scan performed at the 7th week of gestation. A small pseudo‐gestational sac without yolk sac is recognized at the fundus of the uterine cavity.

**FIGURE 3:**
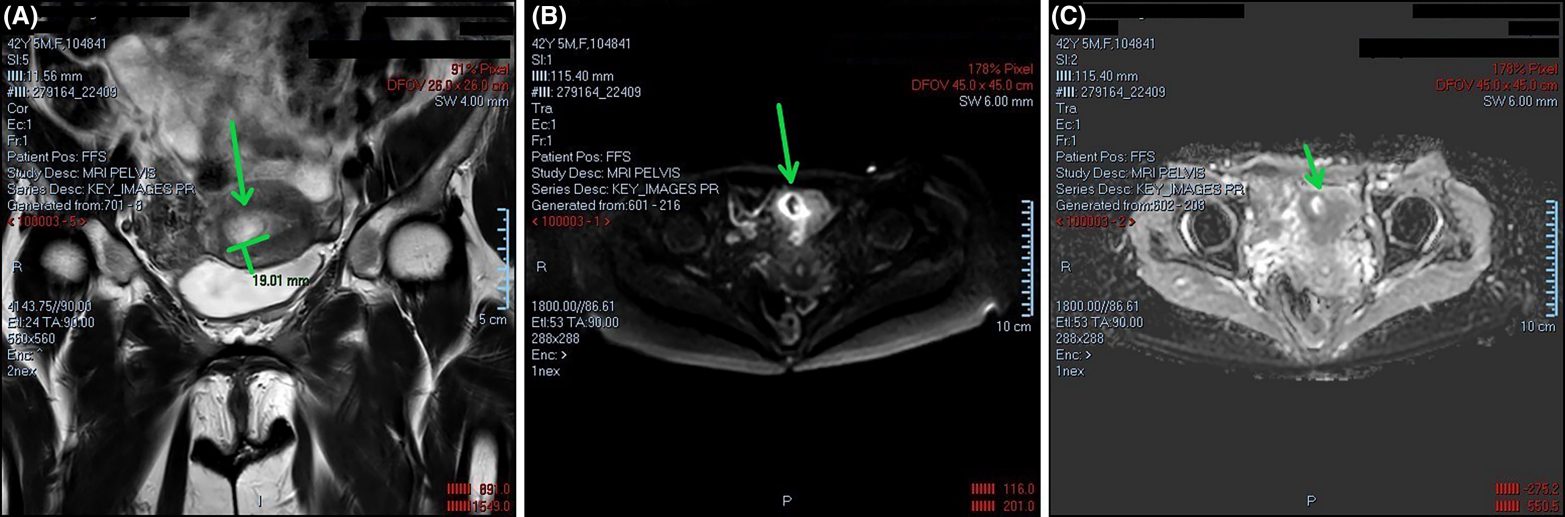
Findings from the MRI scan performed during the 7th week of gestation. The scan verified the diagnosis of cornual pregnancy (A) coronal plane, (B, C) transverse plane.

Given the abnormal β‐hCG levels and the past history of missed abortions, a transvaginal ultrasound was performed during the 6th week of gestation. Ultrasonographic findings included a thick endometrium and a round‐shaped formation at the right uterine cornu, which however possessed no typical features of a gestational sac (Figure 2). Based on ultrasonographic evidence alone, no concrete conclusions could be extracted as to whether the pregnancy was intra‐ or extra‐uterine. Therefore, the patient was advised to and ultimately underwent a Magnetic Resonance Imaging (MRI) scan, which confirmed the diagnosis of cornual ectopic pregnancy, visualized as a 16 by 23 mm region of abnormally increased signal intensity (Figure 3). Following consultation with the patient and discussion of the associated risks of such a pregnancy, the patient consented to undergo hysteroscopic resection of the gestational sac.

**FIGURE 4:**
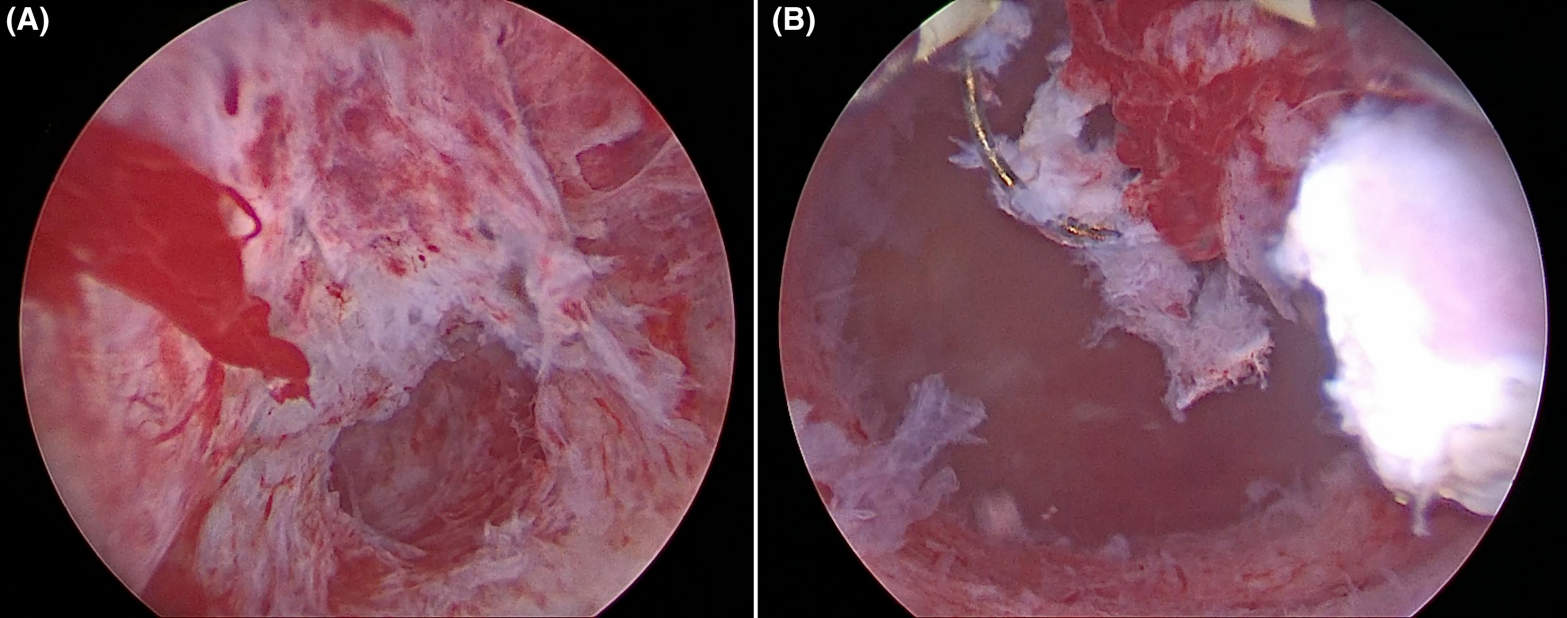
Hysteroscopic resection of ectopic gestatuional sac at the uterine cornu. (A) Gestational sac implanted at the uterine cornu, prior to removal. (B) Gestational sac removed via hysteroscopic diathermy loop without any long‐term effects on uterine anatomy.

**FIGURE 5:**
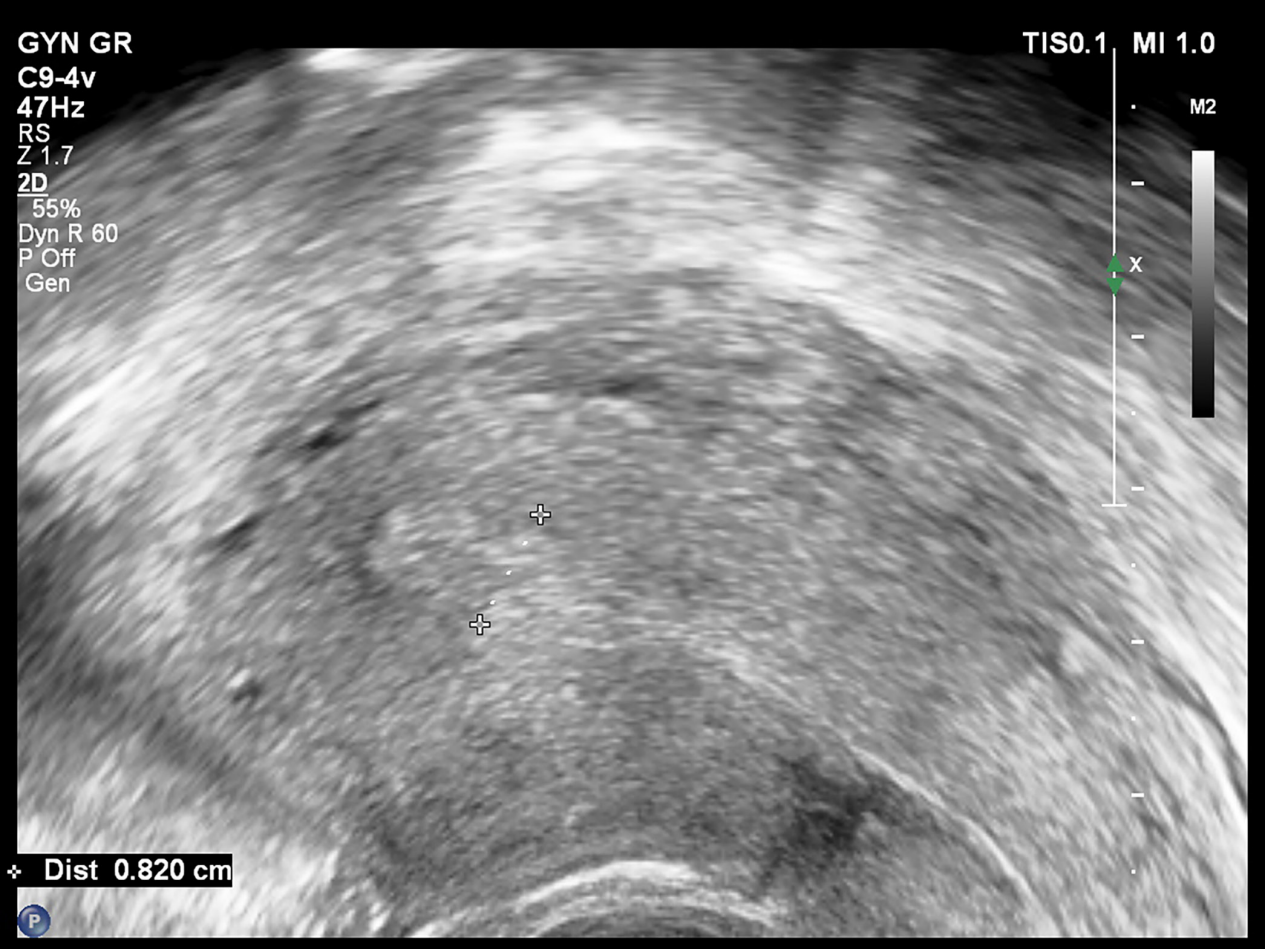
Follow‐up transvaginal ultrasound examination 17 days after hysteroscopic resection of the cornual pregnancy. The ultrasound confirms the absence of any retained products of conception and corroborates the findings from the sequential, diminishing β‐hCG levels.

Pre‐operative β‐hCG levels reached their highest point at 11699 mIU/mL. During the procedure, the cervix was dilated by Hegar dilators up to 9.5 mm. A sorbitol/mannitol solution was used as the distention medium and was infused in the uterine cavity. Adequate infusion pressure was established with the use of a pressure cuff inflated up to 100 mmHg. The gestational sac was hysteroscopically located and resected using diathermy loop (Figure 4). There were no intra‐ or post‐operative complications of note. A measurement of β‐hCG levels 3 days post‐operatively revealed significant decrease, down to 1692 mIU/mL, indicating successful termination of pregnancy and removal of embryonic tissue. Following a thorough assessment, the patient was in good overall condition, reporting only pink spotting and was subsequently discharged. During a follow‐up examination, 2 weeks post‐operatively, the patient was in excellent condition, without any ultrasonographic evidence of prior cornual pregnancy (Figure 5) while her β‐hCG levels were 40 mIU/mL. From that point on, the patient was asked to report any suspicious symptoms and was periodically followed up, until a routine hormonal test 2 months later confirmed that β‐hCG levels had completely subsided and returned to normal.

## 3 DISCUSSION

In this case report, we presented the challenging diagnosis and management of a cornual ectopic pregnancy case. Ectopic pregnancy mortality rate has been documented to be as high as 9–14%, rendering it the leading cause of maternal death in the first trimester of gestation,^3^ while cornual pregnancy In particular may lead to uterine rupture in up to 48.6% of women within the 6th to 26th week of gestation.^5^ Given the aforementioned risk, accurate, timely diagnosis and effective treatment are paramount for the safety of the pregnant woman and in ensuring that she will be able to conceive and gestate again in the future.

In the present case report, while clinical evidence was indicative of ectopic pregnancy, the precise locus could not be located ultrasongraphically. Therefore, MRI scanning was employed as an alternative, which did manage to verify the diagnosis. Kao et al.^6^ in their review describe that cornual pregnancy may be diagnosed via MRI when the gestational sac is identified at the uterine cornu and is surrounded by of an uninterrupted junctional zone that separates it from the endometrium. They additionally stress the need for radiologist to be adequately prepared to diagnose ectopic pregnancy, as ultrasonographic assessment may not always suffice,^6^ similar to our experience during the management of the present case.

Given the increased risk of adverse maternal outcomes, immediate and effective management of cornual pregnancy is paramount. Conservative, pharmacological management with methotrexate administration has been tested as a non‐invasive option for the treatment of cornual pregnancy, via local or systemic methotrexate.^7^ The first reported case of successful resolution of ectopic pregnancy using this methodology was by Tanaka et al.^8^ Since then, multiple similarly successful cases have been published.^9^ Larger case series have also indicated the efficacy of this approach, with Jermy et al.^10^ applying this methotrexate regimen option during the management of 20 cases of ectopic interstitial/cornual pregnancies. They reported successful pregnancy resolution in 94% of cases, they do stress however that this method should be reserved for cases with lower levels of β‐hCG.^10^ Cassik et al.^11^ in their study of 42 women with ectopic interstitial/cornual pregnancy concluded that low levels of initial β‐hCG were the only statistically significant predictor of a final positive outcome, with mean β‐hCG levels in the successful group being 3216 mIU/mL. These conclusions are also corroborated by the latest version of the Royal College of Obstetrician and Gynaecologists guidelines on the matter.^7^ In our case, β‐hCG levels were increasing beyond the levels where conservative management and monitoring would be a safe option; therefore, a more invasive approach was preferred.

The traditional, well‐established, safe approach to cornual ectopic pregnancy is cornual resection via laparotomy or laparoscopy, while hysterectomy may be reserved as a last resort option in life‐threatening cases.^12^, ^13^ Two primary methodologies have been proposed, namely cornuotomy and corneal resection with salpingectomy, both being reported as comparable, with regard to surgical complications and future fertility outcomes.^14^ Regardless of the applied technique, adverse effects on future fertility potential, as well as increased risk of uterine rupture in future pregnancies still remain prevalent risks associated with these methodologies.^1^, ^15^ In a study by Lee et al.^16^ the investigator compared the two approaches and concluded that there were no statistically significant differences between the two approaches apart from operative time (77.11 ± 23.97 min for cornual resection versus 59.36 ± 19.32 min for cornuotomy, *p* = 0.03). No other surgical parameters demonstrated statistically significant differences between the two methods, including no differences in the rate of persistent interstitial pregnancy following treatment.^16^ In our case, since detailed imaging data regarding the sac's location were available and considering the patient's wish to maintain her fertility potential for future attempts, a less radical option was preferred instead.

Hysteroscopic resection of cornual pregnancy is a minimally invasive alternative approach that allows for direct visualization and removal of all the products of gestation, without affecting the rest of the uterus. The first such hysteroscopic resection was reported by Meyer et al.,^17^ performed under laparoscopic guidance. Sanz et al.^18^ further expanded on the concept via hysteroscopy under ultrasonographic guidance and Pal et al.^4^ performed hysteroscopic resection of the gestational sac and subsequently removed the excised products of gestation via suction under combined ultrasonographic and laparoscopic guidance. More recent reports of successful hysteroscopic resection of pregnancy are indicative of the potential of this technique as an alternative with reduced impact on future fertility and maternal outcomes.^19^, ^20^ In our case, MRI data indicated that hysteroscopic removal was a safe and feasible option for this patient, without the need for laparoscopic, more extensive, intervention. Even in settings where detailed imaging guidance is not an option, hysteroscopic removal may be a feasible alternative, provided that adequate ultrasonographic or laparoscopic guidance is available and the patient is very closely monitored for any complications, as was the case with the aforementioned studies that reported on this technique.^4^, ^17^, ^18^ Otherwise, more conventional techniques may be considered.

To our knowledge, this is the first reported case where a combination of transvaginal ultrasound and MRI findings guided the successful hysteroscopic removal of a cornual pregnancy, with the use of a simple resectoscope, without any complications. Given the constant increase in infertility rates, a method that allows for subsequent attempts at conception and pregnancy, without affecting the fertility potential or increasing the risk for uterine rupture during future attempts; such as hysteroscopic resection, seems a promising option. Future research should examine this alternative with larger multi‐center studies and patient series.

## 4 CONCLUSION

Cornual ectopic pregnancy is a rare clinical condition, however very severe and potentially life threatening. Several treatment options are available, however they either entail risks of incomplete treatment (such as with methotrexate administration) or they are associated with adverse effects on future pregnancy and delivery prospects (cornuotomy/cornual resection). Hysteroscopic resection bridges the gap between the available methodologies via ensuring complete removal of all products of gestation, while preserving normal uterine anatomy, however, in our experience, its applications should be exercised with caution and when enough imaging data are available. Larger studies on its efficacy should be conducted in the future in order to further elucidate the place of this methodology in ectopic pregnancy management.

## AUTHOR CONTRIBUTIONS

**Nikolaos Tsagias:** Conceptualization; data curation; formal analysis; funding acquisition; investigation; methodology; resources; supervision; validation; visualization; writing – original draft; writing – review and editing. **Emmanouil M. Xydias:** Data curation; formal analysis; methodology; software; validation; visualization; writing – original draft; writing – review and editing. **Apostolos C. Ziogas:** Data curation; formal analysis; methodology; supervision; validation; writing – original draft; writing – review and editing. **Panagiotis Tsikouras:** Data curation; formal analysis; methodology; resources; validation; writing – original draft; writing – review and editing. **Nikolaos Patsinakidis:** Data curation; investigation; methodology; resources; software; validation; visualization; writing – original draft; writing – review and editing. **Angelos Daniilidis:** Data curation; investigation; methodology; project administration; supervision; validation; writing – original draft; writing – review and editing. **Elias Tsakos:** Conceptualization; funding acquisition; investigation; methodology; project administration; resources; supervision; validation; writing – original draft; writing – review and editing.

## FUNDING INFORMATION

The authors received no financial incentives or compensation for this study.

## CONFLICT OF INTEREST STATEMENT

The authors have no conflict of interest to declare.

## ETHICS STATEMENT

The patient provided informed, written consent for all received treatments and for anonymous publication of findings. Patient care, management and data collection were performed ethically and in accordance with the Helsinki declaration of ethical principles in medical research.

## CONSENT

Written informed consent was obtained from the patient to publish this report in accordance with the journal's patient consent policy.

## Data Availability

All primary research and patient data pertaining to this work are available from the corresponding author upon reasonable request.
